# A study of ethnic, gender and educational differences in attitudes toward COVID-19 vaccines in Israel – implications for vaccination implementation policies

**DOI:** 10.1186/s13584-021-00458-w

**Published:** 2021-03-19

**Authors:** Manfred S. Green, Rania Abdullah, Shiraz Vered, Dorit Nitzan

**Affiliations:** 1 School of Public Health, University of Haifa, Abba Khoushy 199, 3498838 Haifa, Israel; 2 Statistics Consulting Unit, University of Haifa, Haifa, Israel; 3 World Health Organization, European Region, Copenhagen, Denmark

**Keywords:** Vaccine hesitancy, Ethnic groups, Gender, Education, COVID-19, Arabs, Jews

## Abstract

**Background:**

Vaccines for COVID-19 are currently available for the public in Israel. The compliance with vaccination has differed between sectors in Israel and the uptake has been substantially lower in the Arab compared with the Jewish population.

**Aim:**

To assess ethnic and socio-demographic factors in Israel associated with attitudes towards COVID-19 vaccines prior to their introduction.

**Methods:**

A national cross-sectional survey was carried out In Israel during October 2020 using an internet panel of around 100,000 people, supplemented by snowball sampling. A sample of 957 adults aged 30 and over were recruited of whom 606 were Jews (49% males) and 351 were Arabs (38% males).

**Results:**

The sample of Arabs was younger than for the Jewish respondents. Among the men, 27.3% of the Jewish and 23.1% of the Arab respondents wanted to be vaccinated immediately, compared with only 13.6% of Jewish women and 12.0% of Arab women. An affirmative answer to the question as to whether they would refuse the vaccine at any stage was given by 7.7% of Jewish men and 29.9% of Arab men, and 17.2% of Jewish women and 41.0% of Arab women. Higher education was associated with less vaccine hesitancy. In multiple logistic regression analysis, the ethnic and gender differences persisted after controlling for age and education. Other factors associated with vaccine hesitancy were the belief that the government restrictions were too lenient and the frequency of socializing prior to the pandemic.

**Conclusions:**

The study revealed a relatively high percentage reported would be reluctant to get vaccinated, prior to the introduction of the vaccine. This was more marked so for Arabs then Jews, and more so for women within the ethnic groups. While this was not a true random sample, the findings are consistent with the large ethnic differences in compliance with the vaccine, currently encountered and reinforce the policy implications for developing effective communication to increase vaccine adherence. Government policies directed at controlling the pandemic should include sector-specific information campaigns, which are tailored to ensure community engagement, using targeted messages to the suspected vaccine hesitant groups. Government ministries, health service providers and local authorities should join hands with civil society organizations to promote vaccine promotion campaigns.

## Background

Now that vaccines for COVID-19 are becoming available to large sections of the public in many countries, one of the concerns is the uptake of the vaccine by the public [1]. Since these are new vaccines, using accelerated protocols, it is natural that the public may view the safety and efficacy issues with suspicion. This is compounded by the recent increase in the phenomenon of vaccine hesitancy, which is defined as “a reluctance or refusal to be vaccinated or have one’s children vaccinated” [2, 3]. In 2019, the World Health Organization (WHO) ranked vaccine hesitancy among the ten most important threats to global [4].

There is evidence that a significant portion of the population in many countries, may not be willing to get vaccinated against COVID-19 [5, 6]. This will clearly pose a challenge to controlling the COVID-19 pandemic. This has led to the suggestion that there should be vaccine delivery strategies to generate demand [6], which includes engaging trusted sources of authority to advocate for vaccination. It has been suggested that factors related to implementation are likely to contribute more to the success of vaccination programs than the vaccine’s efficacy [7]. Leaders of the health system will need to increase their efforts to promote public confidence in COVID-19 vaccines [6].

Vaccine hesitancy continues to be encountered worldwide, for a variety of vaccines. Outstanding examples include resistance to polio vaccine in Nigeria in 2003 [8], measles vaccine in Europe and North America [3, 9] and the influenza vaccine in 2009 [10]. A number of factors may be responsible for vaccine hesitancy, including government mistrust, fear of side-effects and misinformation [11]. Development of the COVID-19 vaccine has spawned an enormous amount of misinformation about the vaccine [12]. This misinformation has been found to negatively affects people’s self-reported willingness to get vaccinated against the virus and to recommend the vaccine to vulnerable friends and family [13]. The data on ethnic and gender differences in the willingness to be vaccinated against COVID-19 are limited.

Israel has been a leader in the COVID-19 vaccination campaigns, with more than half the adult population having already received at least one dose. However, since the introduction of the COVID-19 vaccine in December 2020, the uptake has been much lower in the Arab population than in the Jewish population and somewhat less in young women [14]. According to the Ministry of Health, by February 14, 2021, 86% of the Jewish population had received at least one dose of the vaccine compared with 51% of the Arab population in the same age group [14]. The aim of this study was to determine ethnic, gender and education differences in Israel in the attitude towards the new COVID-19 vaccines, prior to the introduction of the vaccines. This information is important for developing policies for promoting uptake of the vaccine in different communities.

## Methods

### Study design

This was a national cross-sectional study carried out towards the end of October 2020.

### Target population

The general Israeli population age 30 years and older.

### Sampling

The sampling was conducted by a company conducting internet surveys (iPanel). Based on a panel of over 100,000 participants The Arab sample was augmented with a sample using Facebook. A sample of 957 adults, aged 30 and over, completed the questionnaires online, of whom about 606 were Jews and 351 were Arabs. Since the survey company did not have enough Arabs in their panel of the older groups, we supplemented it by about 50% by a Facebook sample conducted among a large group of Facebook users as a snowball sample.

### The questionnaire

The participants were asked to complete a questionnaire containing 40 questions, including demographic details and their attitudes towards the new COVID-19 vaccines. Some were on a Likert scale of 1 to 5 and others were dichotomous (yes or no). The outcome variables related to the vaccine were based on the following questions:

- If the Ministry of Health approves the trial of a new COVID-19 vaccine, will you be willing to participate in the trial against corona (Yes at all stages = 1 to Not at all = 5)?
- If a vaccine against corona virus is approved, will you be willing to get vaccinated (Yes immediately = 1 to Not at all = 5)?
- If the number of corona cases continues to rise significantly, would you be willing to receive the new vaccine (Yes immediately = 1 to Not at all = 5)?

### The main predictor variables were based on the following questions

- Age (years), gender, education (12 years or professional training, academic degree), ethnicity (Jew, Arab)
- How often do you go out for socializing before the pandemic (1–5)?
- Are the steps the government is taking too lenient (1–5)?
- To what extent is the pandemic dangerous (1–5)?

The questionnaires were tested for face, content and consensual validity. They were translated and back-translated from English to Arabic and Hebrew with the English version being the standard.

### Sample size

The sample size was chosen to give 95% confidence intervals of about + 4% for a prevalence of 50%. For lower prevalence, the interval would be smaller. For sub-groups of Jews and Arabs of different size, the confidence intervals may differ in width. For differences in the prevalence between two groups, for a prevalence of 20% in one group and 30% in the second group, with a significance level of 0.05 and a power of 80%, the required sample sizes should be about 200 in one group and about 600 in the other. This is close to what we achieved for the two ethnic groups.

### Statistical analyses

Descriptive analyses included percentages with confidence intervals. The comparisons of the prevalence between groups were carried out using chi-square tests. Multiple logistic regression analyses were used to examine associations between the outcome variables and ethnic groups, the genders and education, while controlling for the potential confounding variables. Since we were concerned about an interaction between the genders and the ethnic groups, we carried out the regression analysis separately for males and females, and again separately for each ethnic group.

### Ethical approval

The study was approved by the ethics committee of the Faculty of Social Welfare and Health Sciences of the University of Haifa.

## Results

The demographic details of the study population are shown in Table 1.

**Table 1:** Demographic characteristics by gender and ethnic group

	Males			Females		
	Jews *N* = 297	Arabs *N* = 134	*P*. value	Jews *N* = 309	Arabs *N* = 217	*P*. value
**Age (years)**	51.9 ± 15.3 [50,30–85]	41.0 ± 11.3 [37,30–88]	< 0.0001	49.6 ± 12.7 [50,30–79]	42.2 ± 9.5 [40,30–65]	< 0.0001
**Family status**						
Single	51 (17.2)	31 (23.1)	0.1444	84 (27.2)	50 (23.0)	0.2830
In a relationship	246 (82.8)	103 (76.9)		225 (72.8)	167 (77.0)	
**Number of children**	2.8 ± 1.9 [3,0–13]	1.7 ± 1.7 [1.5,0–8]	< 0.0001	2.7 ± 1.9 [3,0–11]	2.5 ± 1.9 [2,0–13]	0.4464
**Education**						
12 years or professional training	142 (47.8)	42 (31.3)	0.0014	145 (46.9)	65 (30.0)	< 0.0001
Academic degree	155 (52.2)	92 (68.7)		164 (57.1)	152 (70.0)	
**Religiosity**						
Not religious/traditional	232 (78.1)	110 (82.1)	0.3454	249 (80.6)	171 (78.8)	0.6162
Religious	65 (21.9)	24 (17.9)		60 (19.4)	46 (21.2)	

Note: Categorical data are reported as n (%) and continuous variables reported as mean ± SD [median, range]

The average age for males was 51.9 for Jews and 41.0 for Arabs (*p* < 0.0001). For females the average age was 49.6 for Jews and 42.2 for Arabs (*p* < 0.0001). The percent with an academic degree for males was 52.2 for Jews and 68.7 for Arabs (*p* = 0.001), and for females it was 57.1 for Jews and 70.0 for Arabs (p < 0.0001).

**Fig. 1:**
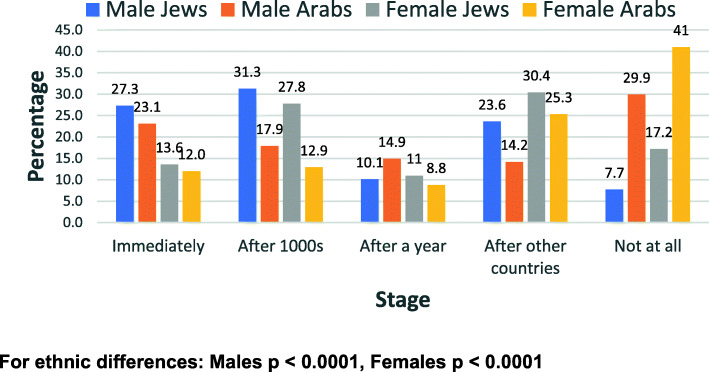
Willingness to receive the COVID-19 vaccine - by gender and ethnic group

**Fig. 2:**
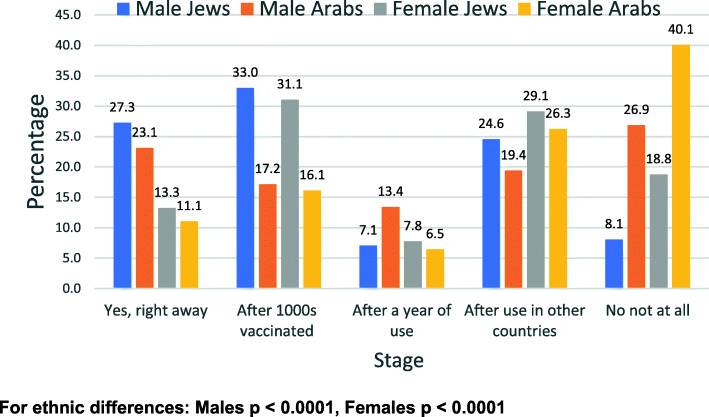
Willingness to be vaccinated if cases increase significantly - by gender and ethnic group

**Fig. 3:**
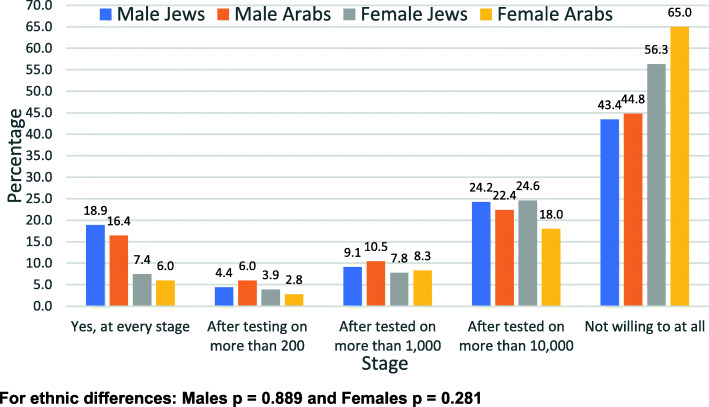
Willingness to take part in a COVID-19 vaccine trial – by gender and ethnic group

**Fig. 4:**
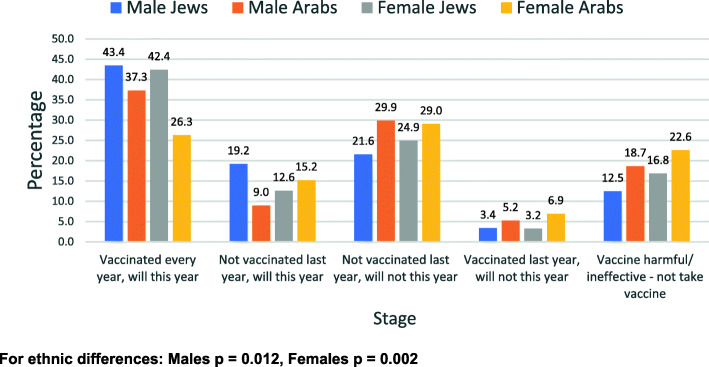
Compliance with influenza vaccine - by gender and ethnic group

Responses to the questions related to the vaccine by gender and ethnic group are shown in all categories (1 to 5) in Figs. 1, 2, 3, and 4.

Figure 1 shows the distribution of the willingness to receive the COVID-19 vaccine, Fig. 2 shows the willingness to be vaccinated if cases increase significantly, Fig. 3 shows the willingness to take part in a COVID-19 vaccine trial and Fig. 4 shows the previous compliance with influenza vaccines. It is clear from the figures that there are marked differences between Arabs and Jews and males and females. The differences are clearest at the extremes of the scales. As regards the willingness to take the vaccine (Fig. 1), the largest gender differences are the willingness to take the vaccine immediately whereas the largest ethnic differences are in the refusal to take the vaccine at all. This pattern is similar for the willingness to take the vaccine if the number of cases increases significantly (Fig. 2). As regards the willingness to take part in a vaccine trial (Fig. 3), the differences are largely between males and females, where males were more likely to volunteer for vaccine trials in both ethnic groups. As regards the previous compliance with influenza vaccines (Fig. 4), the main observation is that Arab women were least likely to take the influenza vaccine.

The answers to the questions as dichotomous variables (combining categories) are shown in Table 2.

**Table 2:** Responses to the questions by gender and ethnic group

		Jews N (%)	Arabs N (%)	Total N (%)	PR (Reference Jews) [95%CI]	p
**Would you want the COVID-19 vaccine immediately**						
**Males**	**Yes**	81 (27.3)	31 (23.1)	112 (26.0)	1.18 [0.82–1.69]	0.365
	**No**	216 (72.7)	103 (76.9)	319 (74.0)		
**Females**	**Yes**	42 (13.6)	26 (12.0)	68 (12.9)	1.13 [0.72–1.79]	0.588
	**No**	267 (86.4)	191 (88.0)	458 (87.1)		
**Would you refuse the COVID-19 vaccine completely**						
**Males**	**Yes**	23 (7.7)	40 (29.9)	63 (14.6)	0.26 [0.16–0.42]	< 0.0001
	**No**	274 (92.3)	94 (70.1)	368 (85.4)		
**Females**	**Yes**	53 (17.2)	89 (41.0)	142 (27.0)	0.42 [0.31–0.56]	< 0.0001
	**No**	256 (82.8)	128 (59.0)	384 (73.0)		
**If the number of cases increased, would you want the COVID-19 vaccine immediately**						
**Males**	**Yes**	81 (27.3)	31 (23.1)	112 (26.0)	1.18 [0.82–1.69]	0.365
	**No**	216 (72.7)	103 (79.9)	319 (74.0)		
**Females**	**Yes**	41 (13.3)	24 (11.1)	65 (12.4)	1.20 [0.75–1.93]	0.449
	**No**	268 (86.7)	193 (88.9)	461 (87.6)		
**Would you be prepared to take part in a COVID-19 vaccine trial at every stage?**						
**Males**	**Yes**	56 (18.9)	22 (16.4)	78 (18.1)	1.15 [0.73–1.18]	0.543
	**No**	241 (81.1)	112 (83.6)	353 (81.9)		
**Females**	**Yes**	23 (7.4)	13 (6.0)	36 (6.8)	1.24 [0.64–2.39]	0.516
	**No**	286 (92.6)	204 (94.0)	490 (93.2)		
**Do you refuse to be vaccinated against influenza this year?**						
**Males**	**Yes**	111 (37.4)	72 (53.7)	183 (42.5)	0.70 [0.56–0.86]	0.002
	**No**	186 (62.6)	62 (46.3)	248 (57.5)		
**Females**	**Yes**	139 (45.0)	127 (58.5)	266 (50.6)	0.77 [0.65–0.91]	0.002
	**No**	170 (55.0)	90 (41.5)	260 (49.4)		
**Do you believe that the pandemic is very dangerous to the public**						
**Males**	**Yes**	247 (83.2)	77 (57.5)	324 (75.2)	1.44 [1.24–1.69]	< 0.0001
	**No**	50 (16.8)	57 (42.5)	107 (24.8)		
**Females**	**Yes**	269 (87.1)	143 (65.9)	412 (78.3)	1.32 [1.19–1.47]	< 0.0001
	**No**	40 (12.9)	74 (34.1)	114 (21.7)		

Among the males, 27.3% of Jews and 23.1% of Arabs said they would want the vaccine immediately (*p* = 0.365) and among females, the figures were much lower at 13.6% for Jews and 12.0% for Arabs (*p* = 588). For the response that they would never want the vaccine, the figures were, among the males, 7.7% of Jews and 29.9% of Arabs said they would never want the vaccine (*p* < 0.0001) and among females, the figures were much higher at 17.2% for Jews and 41.0% for Arabs (p < 0.0001). In answer to the question whether they want the vaccine if the cases increase significantly, among the males, 27.3% of Jews and 23.1% of Arabs said they would want the vaccine immediately (p = 0.365). Among females, the figures were much lower at 13.3% for Jews and 11.1% for Arabs (*p* = 0.449). For the response that they would never want the vaccine, the figures for males were 8.1% of Jews and 26.9% of Arabs. For females the figures were much higher at 18.8% for Jews and 40.1% for Arabs.

In answer to the question whether they would agree to participate in vaccine trials, among males, 18.9% of Jews and 16.4% of Arabs said they would agree to participate in trials at every stage (*p* = 0.543). Among females, the figures were much lower at 7.4% for Jews and 6.0% for Arabs (*p* = 0.516). For the response that they would never agree, the figures for males were 43.4% of Jews and 44.8% of Arabs, and for females, 56.3% of Jews and 65.0% of Arabs.

Regarding the question whether they take the influenza vaccine - among the males, 43.4% of Jews and 37.3% of Arabs stated that they get the vaccine regularly. Among females, the figures were 13.3% for Jews and 11.1% for Arabs. The response to refuse to be vaccinated against influenza this year were among the males 37.4% of Jews and 53.7% of Arabs (*p* = 0.002), among females were 45.0% for Jews and 58.5% for Arabs (p = 0.002). In answer to the question to what extent do you think the pandemic is very dangerous to the public, among males, the prevalence for Jews was 83.2% and for Arabs 57.5% (*p* < 0.0001). For females it was 87.1% for Jews and 65.9% for Arabs (p < 0.0001).

**Fig. 5:**
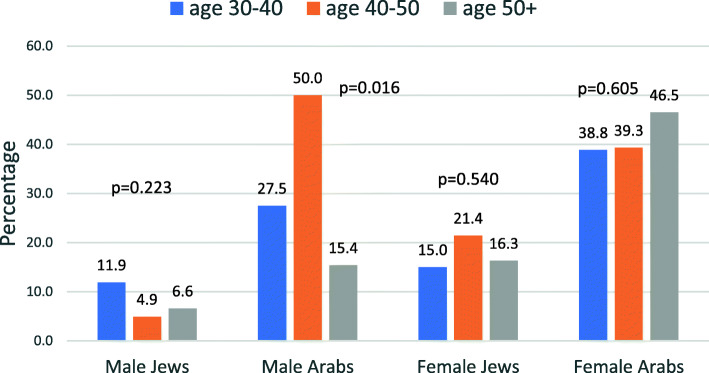
Distribution of respondents who would refuse the vaccine at any stage by gender, ethnic group and age group

The distribution of the responses to the question on refusal to take the vaccine, by sex, ethnic group and three age groups is shown in Fig. 5.

The prevalence rates of those who would refuse the vaccine at all stages were generally higher in the younger age groups except among Arab females, where it was highest in the oldest age group. However, the differences between the age groups were not statistically significant, expect among Arab males, where no trend was clear. Despite some variability between the age groups, the prevalence rates of those who would refuse the vaccine at any stage were almost always higher among Arabs than Jews in both sexes.

The multiple logistic regression analyses are shown in Tables 3 and 4, for males and females separately, to examine the correlates of four questions – do you want the vaccine immediately, would you want the vaccine if cases increased, would you refuse the vaccine totally and would you be willing to participate in vaccine trials.

**Table 3:** Multiple logistic regression analysis of potential correlates of attitudes to the COVID-19 vaccines – by gender

		Prepared to take part in a COVID-19 vaccine trial (Yes vs. No)		Want the vaccine immediately (Yes vs. No)		Refuse the vaccine completely (Yes vs. No)		If cases increased, want the vaccine immediately (Yes vs. No)	
		OR [95%CI]	P. value	OR [95%CI]	P. value	OR [95%CI]	P. value	OR [95%CI]	P. value
**Males**	Age	1.00 [0.98-1.02]	0.929	1.01 [0.99-1.02]	0.293	0.98 [0.96-1.01]	0.117	1.01 [0.99-1.03]	0.222
	Education (Academic degree)	1.41 [0.83-2.39]	0.506	1.39 [0.88-2.18]	0.159	**0.40 [0.22-0.74]**	**0.003**	1.31 [0.83-2.06]	0.247
	Ethnicity (Arab)	0.69 [0.38-1.28]	0.242	0.83 [0.49-1.39]	0.480	**4.79 [2.53-9.06]**	**0.0001>**	0.86 [0.51-1.45]	0.567
	Goes out frequently for socializing (before the pandemic)	1.23 [0.84-1.81]	0.285	1.13 [0.81-1.56]	0.476	**0.58 [0.39-0.88]**	**0.009**	1.22 [0.88-1.70]	0.229
	Believes the steps the government is taking are too lenient	0.83 [0.57-1.19]	0.305	0.78 [0.57-1.08]	0.137	**1.76 [1.17-2.63]**	**0.006**	**0.69 [0.49-0.95]**	**0.025**
	Believes the pandemic is dangerous	**0.52 [0.36-0.75]**	**0.001**	0.80 [0.59-1.09]	0.159	1.08 [0.75-1.57]	0.653	0.76 [0.56-1.03]	0.081
**Females**	Age	1.02 [0.99-1.05]	0.189	1.01 [0.98-1.03]	0.366	0.99 [0.98-1.01]	0.544	1.01 [0.98-1.03]	0.337
	Education (Academic degree)	0.74 [0.36-1.48]	0.393	1.07 [0.62-1.83]	0.812	**0.44 [0.28-0.68]**	**0.0002**	1.25 [0.72-2.18]	0.423
	Ethnicity (Arab)	0.92 [0.41-2.03]	0.830	0.99 [0.56-1.79]	0.997	**3.42 [2.17-5.41]**	**0.0001>**	0.89 [0.49-1.61]	0.690
	Goes out frequently for socializing (before the pandemic)	0.76 [0.47-1.22]	0.254	1.08 [0.75-1.55]	0.679	**0.69 [0.51-0.92]**	**0.013**	1.02 [0.70-1.47]	0.922
	Believes the steps the government is taking are too lenient	0.64 [0.39-1.06]	0.079	0.82 [0.55-1.22]	0.329	1.19 [1.88-1.64]	0.257	0.94 [0.63-1.42]	0.782
	Believes the pandemic is dangerous	1.09 [0.68-1.76]	0.718	**1.44 [2.07-1.00]**	**0.048**	0.86 [0.65-1.14]	0.283	**1.50 [2.18-1.04]**	**0.031**

**Table 4:** Multiple logistic regression analysis of potential correlates of attitudes to the COVID-19 vaccines - by ethnic group

		Prepared to take part in a COVID-19 vaccine trial (Yes vs. No)		Want the vaccine immediately (Yes vs. No)		Refuse the vaccine completely (Yes vs. No)		If cases increased, want the vaccine immediately (Yes vs. No)	
		OR [95%CI]	P. value	OR [95%CI]	P. value	OR [95%CI]	P. value	OR [95%CI]	P. value
**Jews**	Age	1.00 [0.99-1.03]	0.333	1.01 [0.99-1.03]	0.159	0.98 [0.97-1.00]	0.103	1.01 [0.99-1.02]	0.191
	Education (Academic degree)	1.26 [0.77-2.07]	0.357	1.25 [0.83-1.89]	0.288	**0.39 [0.23-0.66]**	**0.0005**	1.34 [0.88-2.03]	0.169
	Gender (Females)	**0.35 [0.21-0.59]**	**<0.0001**	**0.43 [0.28-0.66]**	**<0.0001**	**2.42 [1.42-4.12]**	**0.001**	**0.42 [0.27-0.64]**	**<0.0001**
	Goes out frequently for socializing (before the pandemic)	0.99 [0.67-1.44]	0.936	0.97 [0.71-1.33]	0.865	0.77 [0.53-1.12]	0.169	0.96 [0.70-1.31]	0.786
	Believes the steps the government is taking are too lenient	0.91 [0.59-1.42]	0.683	0.87 [0.60-1.25]	0.445	1.47 [0.90-2.39]	0.123	0.89 [0.62-1.28]	0.526
	Believes the pandemic is dangerous	0.75 [0.50-1.12]	0.158	1.21 [0.87-1.69]	0.266	0.75 [0.49-1.13]	0.163	1.18 [0.84-1.65]	0.346
**Arabs**	Age	0.98 [0.95-1.02]	0.383	0.99 [0.97-1.03]	0.902	0.99 [0.97-1.02]	0.805	1.00 [0.98-1.04]	0.732
	Education (Academic degree)	0.76 [0.35-1.64]	0.485	1.25 [0.65-2.41]	0.506	**0.44 [0.27-0.71]**	**0.0008**	1.19 [0.61-2.32]	0.601
	Gender (Females)	**0.37 [0.17-0.78]**	**0.009**	**0.50 [0.28-0.90]**	**0.021**	1.49 [0.92-2.42]	0.102	**0.47 [0.26-0.85]**	**0.013**
	Goes out frequently for socializing (before the pandemic)	1.12 [0.69-1.83]	0.643	1.39 [0.94-2.07]	0.100	**0.58 [0.42-0.79]**	**0.0006**	**1.55 [1.03-2.32]**	**0.034**
	Believes the steps the government is taking are too lenient	**0.64 [0.41-0.98]**	**0.042**	0.78 [0.54-1.12]	0.173	1.28 [0.96-1.72]	0.098	**0.68 [0.47-0.99]**	**0.047**
	Believes the pandemic is dangerous	0.67 [0.43-1.03]	0.068	0.85 [0.59-1.21]	0.372	1.09 [0.83-1.44]	0.508	0.84 [0.59-1.20]	0.341

The most outstanding ethnic difference was in the total refusal of the vaccine, where the Arab participants were much more likely to say they would refuse vaccine than the Jewish participants. This was similar in both males and females. Also, females in both ethnic groups were more likely to refuse vaccine than males. The findings were consistent after controlling for age and education differences. Other factors associated with vaccine hesitancy were lower education, the belief that the government actions were too lenient and the frequency of socializing prior to the pandemic.

## Discussion

In this study of more than 900 participants, which was conducted before the rollout of the COVID-19 vaccine in Israel, there were marked differences by ethnic group, gender and education in attitudes towards the vaccines. Only a minority of the participants indicated that they would want the COVID-19 vaccine immediately, and this was more marked in females than males in both ethnic groups. When asked about whether they would refuse the vaccine at any stage, the prevalence among Arabs was much higher than for Jews, in both males and females, with highest rate observed in Arab females and the lowest in male Jews. When asked the same question of vaccine uptake if the number of cases increased significantly, the responses remained almost unchanged. For the question on willingness to participate in vaccine trials at some stage, there were mainly gender differences, with the lack of willingness more pronounced in females, regardless of ethnic group. The results were consistent after controlling for age and educational status, where low education was also significantly associated with less willingness to take the vaccine. These findings are consistent with the actual uptake of the vaccine. Despite the considerable success of the current vaccination campaign, the uptake in the Arab population has been much less than in the Jewish population.

The study has strengths and limitations. The sampling was based on panel of internet users and therefore cannot be considered to be representative of the Israeli population. The average age of the Arab participants was lower than the Jewish participants in the sample, in part due to the lower age of the Arab population. This may be due to selective participation in internet survey panels. In addition, the percentage of participants with academic education was higher than in the general population, particularly among the Arab respondents. This also may be due to the selection of people volunteering for internet survey panels. There was no way to estimate response rates, since the questionnaires are sent out to the panel, and the survey is ended when the required sample size is reached. However, this method avoids the high non-response rates in telephone surveys, where people contacted are frequently unavailable for the interview. As regards information bias, multiple answer questions are complicated to ask in telephone interviews whereas in internet surveys, respondents are better able to choose the answer they feel. There is also less of a problem of interviewee fatigue encountered in telephone surveys and less impact from interviewer bias. The difference in education between the two ethnic groups was controlled in multiple logistic regression analyses.

In general, the findings in the present study are consistent with studies in other countries, prior to the introduction of the COVID-19 vaccines. For example, the percentage of people reporting that they would refuse COVID-19 vaccination varied from around 20–25% in the United States and Canada [5] to around 33% in Saudi Arabia [15]. In a study from the UK, 27% stated that they were unsure and 9% said they were unlikely to be vaccinated [16]. In an online survey in the UK and Turkey, 31% of the participants in Turkey and 14% in the UK were unsure about getting themselves vaccinated for a COVID-19 vaccine [17]. In both countries, 3% of the participants said they would refuse vaccination. Studies in representative samples of the French population 18 years of age found that almost a quarter of respondents would refuse vaccination [18, 19]. In a study of Maltese family physicians and their trainees [20], almost two thirds of GPs indicated that they were likely to take the COVID-19 vaccine compared with only a third of trainees, compared with 77% in French healthcare workers [21].

In the current study, approximately 25% were prepared to participate in a clinical trial, more so in males than females, regardless of ethnic group. This compares with the French study, where about 47.6% stated that they will certainly or probably agree to participate in a COVID-19 vaccine clinical trial [19]. In that study, they also found that older age, male gender increased the likelihood of participating in a trial. In addition, they found that being a healthcare worker and individual perceived risk were associated with potential acceptance to participate in a COVID-19 vaccine clinical trial. As in our study, vaccine hesitancy was associated with refusal for participation in a COVID-19 vaccine clinical trial.

The ethnic differences in the willingness to be vaccinated with the COVID-19 vaccine are interesting. For routine childhood vaccines in Israel, the compliance is significantly higher in the Arab population. Thus, the findings of a lower willingness among the Arab respondents to take the COVID-19 is of particular interest. These findings are consistent with the lower vaccination rates now being encountered in the Arab population. This may partly be explained by the fact that childhood vaccines differ from the COVID-19 vaccines in that have been well-integrated into the health system, and childhood vaccines are by and large trusted by the public. However, the novelty of the COVID-19 vaccines and their rapid development has attracted much more than the usual amount of misinformation in the media. The Arab population is quite naturally exposed to the media from Arab-speaking countries and there appears to be considerable misinformation from these sources. There is also an interaction with gender. While women in general showed less willingness to take the COVID-19 vaccine than men, this is particularly evident among Arab women, This could be related to the misinformation that is being spread in the media regarding the potential effect of the vaccine on fertility.

The finding in our study of greater vaccine hesitancy in minorities has been reported from other countries. For example, in Scotland, the percentages of people desiring to be vaccinated were higher among people of white ethnicity as compared with Black, Asian, and minority ethnic groups [22]. In the United States, in a study of nursing homes, willingness to receive the vaccine was associated with white race [23] and this was also observed in population surveys, where African Americans showed less desire to be vaccinated [24–26]. As in our study, male sex and older age have also been found to be associated with a greater willingness to be vaccinated. This has been reported from countries like the United States (), Saudi Arabia and Malta [15, 20]. Similar findings were found in France Nurses and assistant nurses were less likely to get vaccinated than physicians [16]. In our study, we found that higher education was associated with a greater likelihood of wishing to be vaccinated. This is consistent with findings from countries as diverse as the US, Scotland [17] and Saudi Arabia [14].

An important question is why we are seeing the ethnic, gender, age and education differences in vaccine hesitancy. There are many possible factors that differ between groups, including include fear about COVID-19, individual perceived risk [14, 16], perception of the severity of the disease, [20], fear of side-effects and concern about the efficacy and length of testing [9, 11, 27, 28]. Concerns about rushed vaccine development appears to be an important factor that reduces the desire to be vaccinated [19]. Other factors include concerns about commercial interests by pharmaceutical companies and the desire for more evidence on the safety of the vaccine. In France, attitudes to the vaccine were correlated significantly with political partisanship and engagement with the political system [18]. The rift seems to be between people who feel close to governing parties on the one hand and those closer to opposition parties. In the United States, a vaccine that originated from a non-US country was associated with a lower probability of choosing a vaccine [29]. In the study in the UK and Turkey, belief in the natural origin of the virus significantly increased the odds of COVID-19 vaccine acceptance [17]. Intention to be vaccinated has also been associated with having been vaccinated for influenza and individual perceived risk [14, 19]. In a study of vaccine hesitancy in Israel during the influenza pandemic of 2009–10, Velan et al. [10] found that reasoned assessment of risk played a major role in non-compliance with vaccination and the public did not appear to accept government recommendations unconditionally, for which they coined the term “trusting-reflective-non-complier” [10]. They found that nearly a third of the non-vaccinated responders provided reasoned arguments for based mainly on assessment of threat versus actual risk [10]**.**

A number of factors have been described as being important in addressing the issue of vaccine hesitancy. The source of information on vaccines is critical. For example, there is evidence that in Spain, information on vaccines are usually obtained from sources related to the government, professional associations and scientific companies, confirming the central role of government institutions as journalistic sources [30]. These were followed by university scientists, scientific journals and clinicians. Others have noted that an important factor that should be taken into account in vaccination information campaigns are ethnic and gender differences in negative as well as positive emotions [31]. These include attending to negative emotions such as fear and anxiety, raising awareness of emotional manipulations by anti-vaccine disinformation efforts, and activating positive emotions such as altruism and hope as part of vaccine education endeavors [31]. In Italy, health engagement is positively related to the intention to vaccinate and that this relationship is partially mediated by the general attitude towards vaccines [32]. A significantly negative association between religiosity and COVID-19 vaccination intention has been observed which appears to be partially mediated by external high locus of control [33].

Public belief in misinformation about COVID-19 and the COVID-19 vaccines is a major problem. It is likely to be one of the major causes of COVID-19 vaccine hesitancy in Israel, particularly in the Arab population, where there is much more exposure to foreign media where vaccine hesitancy is common. There are many who view this type of misinformation as highly reliable [7]. It is essential that the public be guided to sources providing accurate information. For example, in one study among HIV patients, social service and healthcare providers were the most trusted sources [34]. It has been suggested that vaccine hesitancy among religious minorities may be addressed by better understanding of vaccine decision-making and addressing vaccine hesitancy as part of other public health issues disproportionately affecting minority groups [35, 36].

## Conclusions

The results of this study have important policy implications, particularly in view of evidence of the considerable COVID-19 vaccine hesitancy that is currently being encountered in many countries including Israel. It is becoming increasingly clear that there are marked ethnic and gender differences in the willingness to be vaccinated against COVID-19. The findings have been presented in Israeli government committees for the control of the pandemic, thus assisting decision-makers in identifying sub-groups who are more resistant to vaccination and producing more targeted information campaigns. While this study did not address potential interventions that could be used, these findings are a warning signal to the authorities to focus information campaigns on sensitivity to the ethnic and gender differences in the attitudes towards the vaccine. This will require in-depth research into the ethnic, gender and education related factors associated with COVID-19 vaccine hesitancy. The role of misinformation and how it spreads through the conventional and social media must be identified and carefully assessed. These findings also demonstrate the importance of obtaining valid data early in the planning process in order to tailor information campaigns to address the concerns of different sub-groups in the population. This will also require more community engagement to develop targeted messages to the suspected vaccine hesitant groups. Government ministries, health service providers and local authorities should join hands with civil society organizations to promote vaccine promotion campaigns in the different sectors.

## Data Availability

All data can be made available for inspection.
